# Preclinical and Clinical Studies for Sodium Tungstate: Application in Humans

**DOI:** 10.4172/2155-9899.1000285

**Published:** 2015-01-21

**Authors:** Romina Bertinat, Francisco Nualart, Xuhang Li, Alejandro J. Yáñez, Ramón Gomis

**Affiliations:** 1 Instituto de Bioquímica y Microbiología, Universidad Austral de Chile, Valdivia, Chile; 2 Centro de Microscopía Avanzada, CMA-Bío Bío, Universidad de Concepción, Concepción, Chile; 3 Division of Gastroenterology, Department of Medicine, The Johns Hopkins University School of Medicine, Baltimore, United States; 4 CIBER de Diabetes y Enfermedades Metabólicas Asociadas (CIBERDEM), Barcelona, Spain; 5 Diabetes and Obesity Research Laboratory, IDIBAPS, Barcelona, Spain; 6 Department of Endocrinology and Nutrition, Hospital Clinic, Barcelona, Spain; 7 Faculty of Medicine, University of Barcelona, Barcelona, Spain

**Keywords:** Diabetes, Metabolic syndrome, Hyperglycemia, Weight gain, Intestinal glucose absorption, Gluconeogenesis, Glycogen, Insulin, ERK1/2

## Abstract

Diabetes is a complex metabolic disorder triggered by the deficient secretion of insulin by the pancreatic β-cell or the resistance of peripheral tissues to the action of the hormone. Chronic hyperglycemia is the major consequence of this failure, and also the main cause of diabetic problems. Indeed, several clinical trials have agreed in that tight glycemic control is the best way to stop progression of the disease. Many anti-diabetic drugs for treatment of type 2 diabetes are commercially available, but no ideal normoglycemic agent has been developed yet. Moreover, weight gain is the most common side effect of many oral anti-diabetic agents and insulin, and increased weight has been shown to worsen glycemic control and increase the risk of diabetes progression. In this sense, the inorganic salt sodium tungstate (NaW) has been studied in different animal models of metabolic syndrome and diabetes, proving to have a potent effect on normalizing blood glucose levels and reducing body weight, without any hypoglycemic action. Although the liver has been studied as the main site of NaW action, positive effects have been also addressed in muscle, pancreas, brain, adipose tissue and intestine, explaining the effective anti-diabetic action of this salt. Here, we review NaW research to date in these different target organs. We believe that NaW deserves more attention, since all available anti-diabetic treatments remain suboptimal and new therapeutics are urgently needed.

## Introduction

The control of energy homeostasis and blood glucose concentrations depends on the exquisite coordination of the function of several organs and tissues, among them the liver, muscle, fat, pancreas and the brain. Some of these organs and tissues have major roles in the use and storage of nutrients in the form of glycogen or triglycerides and in the release of glucose or free fatty acids into the blood in periods of metabolic needs, and they all participate in imbalanced homeostasis during metabolic disorders [1,2]. Diabetes is a progressive metabolic disease characterized by chronic hyperglycemia and disturbances of carbohydrate, fat, and protein metabolism [3]. Diabetes is triggered by the deficient secretion of insulin by the pancreatic β-cell (T1D) or the resistance of peripheral tissues to the action of this hormone (T2D). T1D affects 10%, whereas T2D affects 90% of diabetic patients, and overall they account for 382 million people worldwide [4]. Chronic elevation of blood glucose levels is the major consequence of insulin deficiency or resistance, and also the main cause of long-term diabetic problems [5,6]. Tight glycemic control is widely recognized as the best method to delay diabetic progression and complications [7–9], but an increased risk of severe hypoglycemia comes with lower blood glucose levels [10–12]. Furthermore, most T2D patients are overweight, adding another risk factor for increased morbidity and mortality [12,13].

T1D patients require exogenous insulin administration early in the development of the disease and a lifelong treatment is required [11]. T2D patients need insulin supplementation in advanced stages of the disease, when β-cells no longer compensate peripheral insulin resistance. Despite new formulations have been developed, insulin injection presents always a higher risk of hypoglycemic events [11,14,15]. T2D is early treated with one or a combination of the following drugs: a) Sulfonylureas (SUs) were the first drugs used for treatment of T2D. SUs lower blood glucose levels by stimulating insulin secretion from β-cells, but they are associated with a progressive decline in β-cell function. Hypoglycemia and weight gain are common effects of SUs therapy [13,16]. b) α-glucosidase inhibitors are competitive inhibitors of pancreatic α-amylase and intestinal α-glucosidase in the intestine and delay glucose absorption. Limited gastrointestinal tolerability has restricted their use [16]. c) Glinides are insulin releasers that stimulate prandial insulin secretion. They are associated with weight gain and hypoglycemic events [11,13,16]. d) Thiazolidinediones are insulin-sensitizers that improve insulin sensitivity through gene regulation, and weight gain has also been associated with their administration [13,16]. e) *Biguanides*, mainly represented by metformin, exert their anti-diabetic action through inhibition of hepatic gluconeogenesis and opposing the action of glucagon [17], but special considerations must be taken when administered to patients with renal and/or hepatic impairment [16]. f) Dipeptidyl peptidase-4 inhibitors (DPP-4) decrease the inactivation of incretins to stimulate insulin release in a glucose-dependent manner [18]. Most DPP-4 inhibitors are eliminated through the kidney, so the same cautions as for metformin must be taken into account [18]. g) Inhibitors of the main mediator of glucose reabsorption by the kidney, sodium-glucose cotransporter isoform 2 (SGLT2), have also shown their great potential [19]. A more complete analysis of the mechanism of action and side effects of these and other anti-diabetic drugs is out of the scope of this review. Nevertheless, it is worthy to note that anti-diabetic drugs receive marketing authorization if they reduce glycosylated hemoglobin (HbA1c) levels, which are in relation with blood glucose concentrations [15,20]. However, it has been shown that anti-diabetic drugs reduce HbA1c without a reduction in clinical events, and based on trial evidence Boussageon et al. [15] have proposed that “the clinical efficacy of anti-diabetic drugs is disappointing and does not support the millions of prescriptions being written for them”. Hence, given that hypoglycemia and weight gain are still key limiting steps in maintaining normoglycemia in untreated or treated diabetic patients, finding and developing new highly effective anti-diabetic agents is of urgent need for diabetic care.

## Efect of Sodium Tungstate (Naw) In Experimental Animal Models

Tungstate is the oxoanionic form of tungsten, a transition metal also known as wolfram [21]. Tungstate presents a tetrahedral structure and it is a phosphate analogue, being widely used as a phosphatase inhibitor [22–24], although this property is not enough to account for all the anti-diabetic actions reported to date [25]. Sodium tungstate (NaW) has shown to be an effective and safety normoglycemic agent in different T1D and T2D experimental animal models, being administered orally (*ad libitum*, in tap water) as a non-invasive easy-to-use anti-diabetic agent [26,27]. NaW has proven to effectively normalize blood glucose [26–29] and triglycerides levels [28,30,31], to restore hepatic glucose metabolism [26,27,29], to stimulate pancreatic [32–35], cardiac [36] and sexual [37,38] function, to reduce blood pressure [39–41], to exert anti-obesity [28,30,31,42]; and immunomodulatory [43] activities. Notably, NaW does not produce hypoglycemic episodes, and it also stimulates weight loss [31,44]. NaW concentrations that are active *in vitro* are similar to the plasma levels determined in NaW-treated animals [45], and no apparent NaW toxicity has been reported to date neither *in vitro* [25] nor *in vivo* [26,27,29,36,46]. In fact, NaW has also been proposed as an adjuvant in the treatment of cancer [47,48], and a protector in liver necrosis and hepatic failure due to oxidative damage [49,50]. Despite the evidence described above strongly suggest that NaW exerts beneficial effects at the physiological level, the mechanism of action of NaW is more elusive. Most of insulin effects have been mimicked by NaW, but the molecular events seems to differ. We are going to revise how NaW affects the activity of tissues involved in energy homeostasis, and how it improves the metabolic imbalance during insulin resistance and diabetes.

### Liver

Glycogen synthesis and breakdown are among the most important functions of the liver in order to maintain normal blood glucose levels during short-term fasting, and induction of hepatic glycogen synthesis is one of the main anabolic effects of insulin, that is decreased during diabetes, contributing to abnormal extraction of glucose from the blood [51]. Oral administration of NaW to streptozotocin (STZ)-diabetic rats (T1D model) and Zucker diabetic fatty (ZDF) rats (T2D model) stimulated hepatic glucose metabolism, with a common increase in glycogen levels [26,27,29,46]. An increase in glycogen synthase (GS) activity was detected in STZ-diabetic but not in ZDF rats [26,27,29,46].

**Figure 1:**
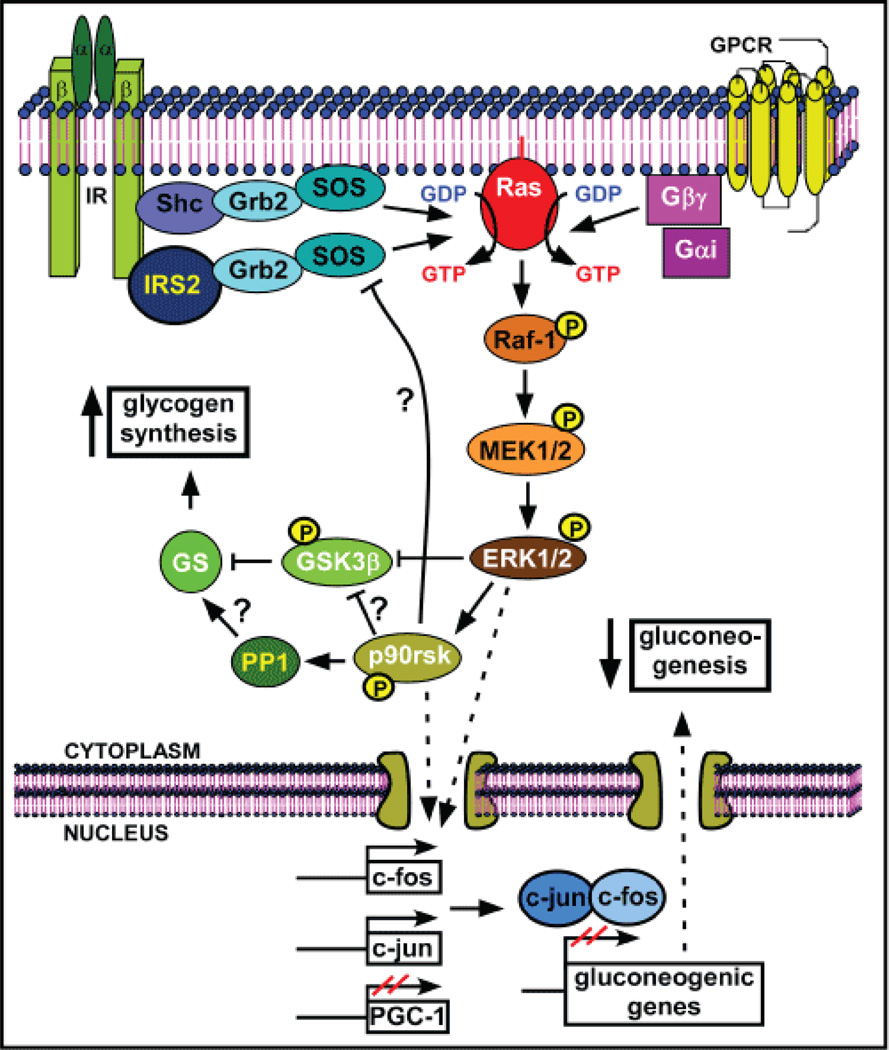
NaW signaling pathway in liver NaW does not directly activate IR, or other tyrosine kinase receptors, such as EGF or IGF receptors in hepatocytes. Rather, it activates Gαi2 and Gβγ subunits of GPCR, to stimulate the activity of the small GTPase Ras, initiating the signaling transduction through sequential phosphorylation of Raf-1, MEK1/2 and ERK1/2, which in turn phosphorylates GSK3β and p90rsk. Phosphorylated GSK3β is inactive and alleviates the inhibitory phosphorylation over GS, favoring activation of glycogen synthesis. Although it has not been demonstrated for NaW action, p90rsk is known to be involved in GSK3β inactivation, PP1 activation and negative feedback upstream in the pathway through inactivation of SOS. PP1 is the main phosphatase activity participating in GS dephosphorylation and stimulation of glycogen synthesis, but the actual participation of NaW in PP1 activation remains to be established. In parallel, NaW-madiated ERK1/2 activation stimulates c-fos and c-jun, and inhibits PGC-1 transcriptional expression through yet unknown mechanisms. Since PGC-1 is a positive modulator, whereas c-fos and c-jun are negative regulators of the transactivation of gluconeogenic genes, NaW action finally induces down-regulation of gluconeogenic enzymes and, hence, the gluconeogenic pathway. In IRS2 KO mice, NaW was unable to exert consistent effects on glycogen synthesis and gluconeogenesis, highlighting the importance of IRS2 in NaW signaling pathway in hepatocytes. **White letter:** proteins are modified after NaW treatment. **Black letter:** proteins involved in the normal pathway, which are expected to participate in NaW signaling, although it has not been determined; **Yellow letter:** evidence supports these proteins as potentially involved in NaW signalling; **?:** regulatory mechanisms that have not been confirmed for NaW signalling; **P:** phosphorylation.

Moreover, normalization of fructose 2,6-bisphosphate (F2,6P2) levels, and restoration of 6-phosphofructo 2-kinase (PFK2); [26,46,52], liver pyruvate kinase (L-PK) and glycogen phosphorylase (GP) activities were observed [26,29], whereas glucokinase (GK) activity and glucose 6-phosphate (G6P) levels were partially recovered [26,29]. Also, mRNA levels of GP, GK and PK [26,27] were enhanced in the liver after NaW treatment of STZ-diabetic rats. A key discovery in the insulin-mimetic activity of NaW was that it by-passes the insulin receptor (IR) to activate GS and to promote glycogen synthesis in isolated rat hepatocytes [25]. Epidermal growth factor (EGF) and insulin-like growth factor (IGF) receptors were not activated either [53]. Notably, NaW had no consistent effect on altered hepatic glucose metabolism in Insulin receptor substrate (IRS)-2 knockout mice, a model of T2D [35], which indicates that IRS-2, and maybe the complete insulin pathway, is required for NaW effects in liver. Like insulin, NaW induced a rapid and transient phosphorylation, i.e activation, of extracellular signal-regulated kinase (ERK) 1/2, without delaying its dephosphorylation [25]. Activation of ERK1/2 was the first molecular event unequivocally related with NaW signaling, and stimulation of metabolic but not mitogenic effects of ERK1/2 in the Chinese hamster ovary (CHO)-R cell line (expressing the wild-type human insulin receptor) strengthened the different signaling mechanisms between insulin and NaW [25]. Zafra et al. [53] have unraveled upstream hepatic NaW signaling involved in ERK1/2 activation in hepatocytes, showing that both Gαi2 and Gβγ subunits of G-proteins participate in its mechanism of action. NaW induced activation of the small GTPase Ras, which in turn induced phoshorylation (activation) of Raf and then mitogen-activated protein kinase kinase (MEK), the last one being the main kinase activity involved in ERK1/2 phosphorylation [53] (Figure 1). NaW induced-ERK1/2 activation triggered phosphorylation of downstream kinases, including 90 KDa ribosomal S6 kinase (p90rsk) and glycogen synthase kinase 3β (GSK3β), without activation of protein kinase B (PKB/Akt) in both isolated hepatocytes [25,27] and rat liver [27]. Remarkably, PKB/Akt has been recognized as the main contributor to insulin induced-GSK3β phosphorylation [54]. The normally active (dephosphorylated) GSK3β is a key player in the insulin pathway, and its inactivation (phosphorylation) is considered essential for a normal insulin response [55]. Inhibition of GSK3β by NaW treatment relieved GS phosphorylation, allowing an increase in glycogen synthetic activity [53] (Figure 1). Activation of p90rsk has been reported to inhibit GSK3β and to feedback inhibition of the Ras-ERK pathway though phosphorylation of the Ras GTP/GDP-exchange factor, Son of sevenless (SOS) [56]. Besides, it has been shown that NaW exerts an inhibitory effect over Gcl7p protein phosphatase in yeast, whose mammalian orthologue is protein phosphatase 1 (PP1), the main phosphatase acting on GS [57].

However, the actual participation of NaW in the in vivo activity of mammalian PP1 remains to be established.

*De novo* synthesis of glucose through gluconeogenesis in human liver is important for maintaining euglycemia in normal conditions [58], but it is abnormally increased in diabetes, being one of the major contributors to chronic hyperglycemia [59,60]. Another well-known metabolic effect of insulin is inhibition of hepatic gluconeogenesis through down-regulation of the expression of gluconeogenic genes [59,60].

NaW treatment has shown to normalize mRNA expression of the three key rate-limiting gluconeogenic enzymes phosphoenolpyruvate carboxykinase (PEPCK), fructose 1,6-bisphosphatase (FBPase) and glucose 6-phosphatase (G6Pase) in the liver from STZ-diabetic rats [26,27], and also PEPCK proteins levels in ZDF [29] and STZ [27] diabetic rats. A potent inhibitory effect of NaW over G6Pase has also been reported [61]. Furthermore, it has been shown that diabetes impairs nuclear translocation of FBPase in rat liver [62–64], which has been suggested as an insulin-induced sequestration mechanism for this gluconeogenic enzyme. NaW administration to fasted and fed STZ-diabetic rats recovered FBPase nuclear accumulation (unpublished data), in the same way as insulin did [62]. Besides, NaW normalized expression levels of the transcription factors c-jun and c-fos, involved in down-regulation of hepatic gluconeogenic genes [65]. Also, despite NaW did not affect expression of the transcription factors hepatic nuclear factor 4 alpha (HNF4α) and Forkhead box protein O1 (FOXO1), both important contributors in regulation of hepatic gluconeogenesis, it did reduce expression of Peroxisome Proliferator-Activated Receptor (PPAR)-γ coactivator 1 alpha (PGC1α), a cofactor required for complete induction of PEPCK and G6Pase expression through HNF4α and FOXO1 transcriptional activity; [59,60,66]. GSK3β has been shown to act as a key regulator for the expression of gluconeogenic genes through HNF4α and FOXO1 [55], being a good candidate in mediating NaW effect on hepatic gluconeogenic activity. Interestingly, the glucocorticoid receptor (GR) enhances the expression of PGC1, which in turn co-activates both GR itself and FOXO1 to induce gluconeogenic gene expression [60,67]. NaW has been shown to interact with GR, blocking its activation by ligand and the consequent ability to bind DNA [68,69]. Moreover, ERK-mediated phosphorylation of GR has been implicated in cytosolic retention and inhibition of GR transcriptional activity [70,71], offering new pathways to explain NaW anti-gluconeogenic actions. Finally and notably, all these NaW effects in liver metabolism were not observed in treated non-diabetic rats [26,27,29,46].

#### Summary

NaW stimulates hepatic glycogen synthesis and inhibits hepatic endogenous glucose production and release contributing to normalization of glycemia in diabetes that is dependent on ERK1/2, but not PKB/Akt activation.

### Muscle

**Figure 2:**
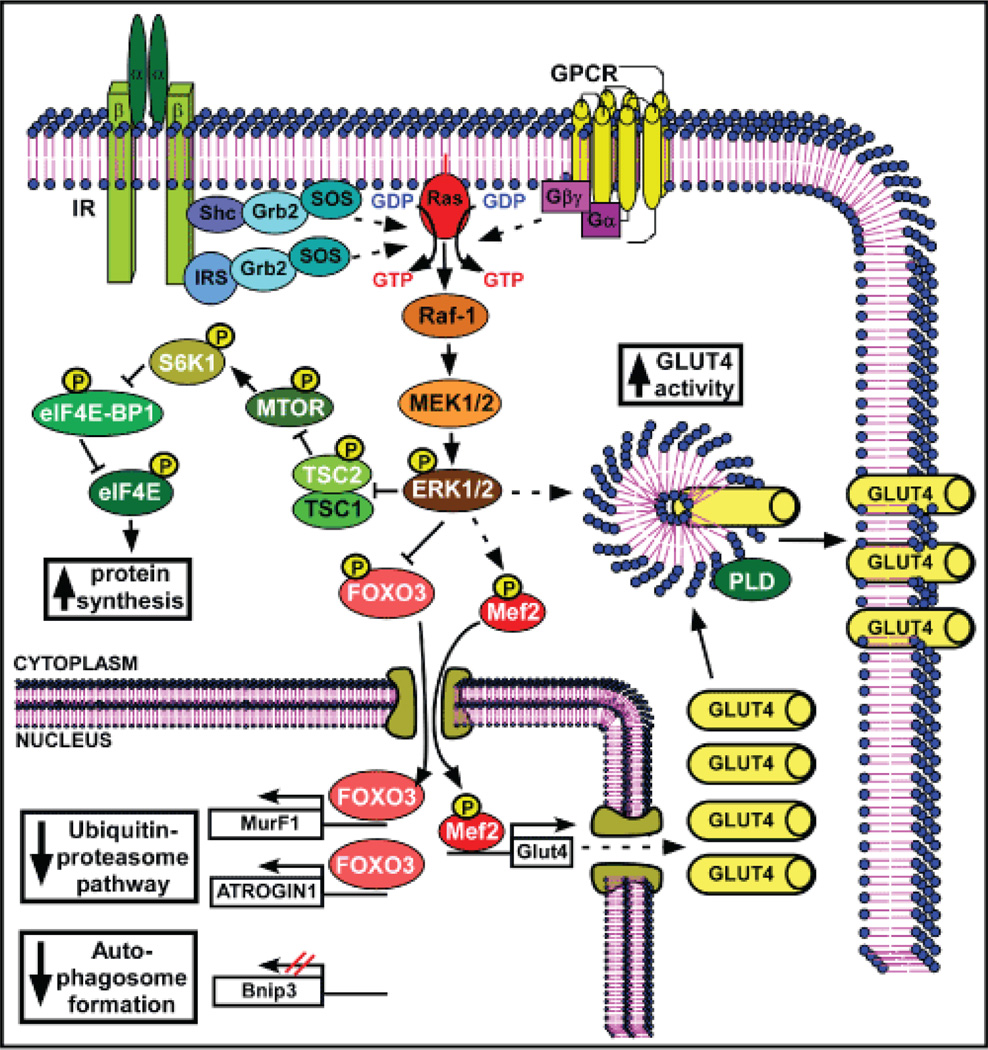
NaW signaling pathway in muscle There is no available information regarding NaW signaling upstream of ERK1/2 in muscle, except that again IR is not activated. The same scheme proposed for liver upstream of ERK1/2 (Scheme 1) was used as a reference. NaW induced-ERK1/2 activation led to phosphorylation and inhibition of TSC2, relieving inhibition on MTOR. Activated MTOR phosphorylates and activates S6K1, which inhibits eIF4E-BP1, alleviating eIF4E inhibition, and promoting protein translation. Moreover, ERK1/2 phosphorylates FOXO3 and Mef2 transcription factors. Phosphorylated FOXO3 is inactive and cannot translocate into the nucleus to transactivate MurF1 and ATROGIN1 genes, down-regulating the expression of these important ubiquitin ligases, and reducing the activity of the ubiquitin-proteasome pathway of protein degradation. Besides, NaW induces a decline in auto-phagosome formation through the down-regulation of Bnip3. On the other hand, phosphorylated Mef2 is active and can enter the nucleus to promote Glut4 gene expression, which together with above-mentioned eIF4E stimulation led to enhanced protein synthesis of GLUT4 transporter. Finally, NaW-induced ERK1/2 activation stimulates fusion of GLUT4-containing vesicles with the plasma membrane, maybe through direct activation of PLD, enhancing GLUT4 activity and glucose uptake. **White letter:** proteins are modified after NaW treatment; **Black letter:** proteins involved in the normal pathway, which are expected to be involved in NaW signaling, although it has not been determined; **P:** phosphorylation.

Muscle is the major site of insulin-dependent glucose uptake, and its ability to remove blood glucose is greatly impaired in the diabetic condition [72]. The main facilitative glucose transporter in muscle is GLUT4, which is normally located in intracellular compartments and rapidly translocates to the plasma membrane in response to insulin, accounting for increased transport activity [73]. GLUT4 expression is decreased in diabetic rats, but NaW has shown to enhance mRNA and protein levels, and also translocation of GLUT4 to the plasma membrane in the diaphragm from NaW-treated STZ-diabetic rats [74] (Figure 2). In vitro, NaW induced an increase in glucose transport and in the amount of GLUT4 mRNA and protein levels in L6 myotubes [75]. NaW upregulated the transcriptional activity of Glut4 gene promoter, and this activation depended on the presence of the myocyte enhancer factor 2 (MEF2) binding site [75] (Figure 2).

Specifically, an increase in the nuclear content of the transcription factors MEF2-A; -B and -D isoforms, together with an enhanced interaction with the promoter binding site, was stimulated by NaW treatment [75]. Moreover, in contrast to insulin, NaW stimulated membrane translocation of GLUT4 independently of IR, phosphatidylinositol-4,5-bisphosphate 3-kinase (PI3K) or PKB/Akt activation. Also, a lack in 5'AMP-activated protein kinase (AMPK) activation was observed in response to NaW or insulin [75].

NaW-induced Glut4 gene expression and increased glucose uptake in L6 myotubes was dependent on ERK1/2 activation, positioning this kinase as a key mediator in the insulin-mimetic effects of NaW in muscle. Besides, phospholipase D (PLD) was also involved in the NaW-induced glucose uptake [75].

Deleterious effects of chronic hyperglycemia trigger accelerated loss of muscle mass [76]. Like insulin, NaW has shown to stimulate protein synthesis in L6 myotubes, counteracting dexamethasone-induced protein degradation [77]. As it has been demonstrated for other cellular effects, NaW action on protein synthesis depends again on ERK1/2, but not PKB/Akt activation [77]. ERK1/2 is involved in phosphorylation and inactivation of tuberin (TSC2) [77], a negative regulator of the mechanistic target of rapamycin (MTOR). NaW also induced phosphorylation (activation) of MTOR, which in turn phosphorylated 70 KDa S6 kinase 1 (S6K1), eukaryotic initiation factor 4E binding protein-1 (eIF4E-BP1) and eIF4E, promoting protein synthesis [77] (Figure 2). In parallel, NaW stimulated phosphorylation (inactivation) and cytosolic arrest, and inhibited dexamethasone-induced transcriptional activity of the Forkhead box O3 (FOXO3) transcription factor, which participates in ubiquitin-proteasome and lysosomal (autophagy) pathways of protein degradation [77]. Indeed, NaW counteracted the dexamethasone-induced ubiquitin promoter-dependent expression of the main ubiquitin ligases, Muscle RING-finger protein 1 (MuRF1) and F-Box Only Protein 3 (ATROGIN1) [77]. Moreover, NaW impaired dexamethasone-induced cleavage and phosphatylethanolamine conjugation of microtubule-associated protein 1 light chain 3 (LC3) and expression of Bcl-2 and nineteen-kDa interacting protein-37 (Bnip3), two key proteins involved in autophagosome formation [77] (Figure 2).

Hyperglycemia is associated with a higher risk of cardiac dysfunction, which in turn results in a high percentage of morbidity and mortality in diabetic patients [78]. Functional abnormalities of diabetic heart are attributed to different cellular alterations, such as reduction in the rate of contraction and relaxation, dysregulation of Ca^2+^ homeostasis and altered ionic currents, all of them mediated, at least in part, through enhanced production of reactive oxygen species (ROS) and oxidative stress [78,79]. NaW has been shown to reduce blood pressure in animal models of hypertension [40,41] and metabolic syndrome [39]. Left ventricular pressure development, the rate of contraction (+dP/dT), and the rate of relaxation (−dP/dT) were significantly enhanced in NaW-treated STZ-diabetic rats [36]. This improvement in contractile deficits elicited by NaW has been attributed to restoration of intracellular levels of free calcium (Ca^2+^) ions and altered ionic currents [79]. Indeed, it has been shown that NaW activates the large-conductance voltage- and Ca^2+^-dependent K^+^ (BK) channel [80,81], and stimulates the release of vasodilator prostanoids from the vascular wall [39], both participating in the control of arterial tone through induction of vasodilatation. Hyperglycemia promotes an abnormal increase in ROS generation through enhanced activity of different enzyme systems, such as the mitochondrial respiratory chain, NADPH oxidase (NOX) and xanthine oxidase (XO) [5,6]. ROS can directly damage lipids, proteins and DNA, and modulate intracellular signaling pathways and redox sensitive transcription factors, altering protein expression. In fact, redox induced-modifications of protein structure and expression have been linked to altered muscle activity [82,83]. Interestingly, it has been demonstrated that myocardial injury involves a xanthine oxidase-dependent mechanism, and that NaW treatment significantly decreased cardiac damage and normalize blood pressure [40,41,84]. This is in agreement with the fact that NaW is a well-known inhibitor of molybdo-enzymes, among them xanthine oxidase [85]. Any effect of NaW on the other enzyme systems involved in exacerbated ROS production remains to be established.

#### Summary

NaW enhances glucose uptake and protein synthesis through activation of ERK1/2, but not PKB/Akt (or PI3K either in the case of glucose uptake), contributing to blood glucose normalization and restoration of muscle mass. Besides, NaW reduces blood pressure through improvement of contractile deficits and decrease of ROS production.

### Pancreas

The β-cell in the Langerhan's islet from the pancreas is the site of production of the hormone insulin [86]. In order to maintain normoglycemia, β-cells secrete insulin in response to different stimuli [87,88]. In T1D, β-cells are selectively eliminated by an autoimmune mechanism [89], whereas insulin resistance of peripheral tissues induces early over-stimulation and later progressive loss of functionality and finally death (mainly through apoptosis) of β-cells in T2D [90]. STZ-induced diabetes produces β-cell death, offering a good model of T1D [91]. A morphometric analysis in diabetic rats showed that NaW treatment one week before STZ induction preserved normal islets volume density, volume-weighted mean islets volume, and mass of β-cells, islets, and pancreas [92], suggesting that NaW protected from STZ-induced damage. Moreover, blood and pancreas antioxidant power increased, whilst lipid peroxidation decreased in NaW-treated diabetic rats [93]. NaW treatment after STZ-induced diabetes in rats or in IRS-2 knockout diabetic mice significantly decreased β-cell apoptosis, and also increased β-cell replication, explaining total increase in β-cell mass [32,35]. The IRS-2 knockout mouse is a good model for the study of islet survival because most animals by the 8^th^ week of life start losing β-cell mass due to apoptosis [35]. IRS-2 participates in insulin- and other growth factors-mediated cell survival and proliferation, and NaW treatment has shown to decrease upregulated expression of several genes involved in the mitochondrial apoptotic pathway and inflammatory response in islets lacking IRS-2, indicating that NaW primarily targets β-cell death mechanisms through activation of endogenous kinases [35]. Interestingly, NaW alone induced a little increase in proliferation of the INS-1E rat β-cell line, but when these cells were incubated with serum from NaW-treated diabetic rats, the rate of proliferation was greatly enhanced, suggesting that the proliferative effect of NaW on the pancreatic β-cell is through indirect mechanisms [32]. A transcriptomic analysis of pancreas from STZ-diabetic rats has revealed that the majority of genes whose expression is altered during diabetes, are restored to different degrees by NaW treatment [32]. Among them, Raf kinase inhibitory protein (Rkip) gene expression was upregulated in the diabetic pancreas and normalized by NaW treatment [32], according with an anti-proliferative function of Rkip in β-cells [94]. ERK1/2 has been shown to participate in NaW-induced proliferation of INS-1E cells [32]. Since Rkip inhibits Raf-1 kinase, which phosphorylates MEK, which in turn phosphorylates ERK1/2, NaW-induced normalization of Rkip expression leads to an increase in the MAPK pathway activity, favoring proliferation. Moreover, NaW was able to restore ERK1/2 phosphorylation in islets from diabetic IRS-2 knockout mice [35], suggesting that IRS-2 is not necessary for NaW signaling through the ERK pathway in the β-cell. Also, NaW-induced suppression of upregulated proapoptotic and inflammatory genes in IRS-2 knockout mice was dependent on the activation of the MAPK pathway [35].

In the perfused rat pancreas, NaW induced a dose-dependent stimulation of insulin secretion in the presence of normal (5.5mM) or moderately high (9mM), but not low (3.2mM) glucose concentrations [34], explaining in part the absent hypoglycemic effect reported for NaW treatment. In isolated islets from oral NaW-treated neonatally STZ (nSTZ)-induced diabetic rats, a model of T2D, a partial recovery of insulin content and pre-proinsulin mRNA levels was detected, together with the restoration of β-cell response to an increase in glucose concentration [95]. NaW did not altered glucagon secretion or arginine-induced insulin secretion, and the insulinotropic effect of NaW was abolished by diazoxide, somatostatin and amylin, indicating that its effect was dependent on normal glucose metabolism and stimulation of insulin exocytosis by the β-cell [34]. In the BRIN BD11 rat β-cell line, 300µM NaW also increased insulin content, basal insulin release and the responsiveness to glucose and other secretagogues [96]. In MIN6 mouse β-cell line, 100µM NaW induced transient activation of p38 and PI3K, and downstream PI3K-dependent phosphorylation of S6K1 [97]. Moreover, a selective inhibitor of the prenylation of the Rho-GTPase Rac1/Cdc42, an upstream activator of p38, completely blocked the NaW-induced phosphorylation of p38 in MIN6 cells [97]. p38 phosphorylation has also been observed in isolated islets from NaW-treated diabetic rats [33]. p38 and PI3K are known to mediate high glucose-induced phosphorylation, nuclear translocation and activation of the pancreatic and duodenal homeobox 1 (PDX-1) transcription factor, which is involved in pancreas development, insulin expression and β-cell functionality [98]. NaW stimulated PDX-1 phosphorylation in rat islets both in vitro and in vivo [33], augmenting the number of insulin and PDX-1 positive cells in pancreas from NaW-treated STZ-diabetic rats [32,33]. In MIN6 cells, NaW induced a p38- and PI3K-dependent nuclear translocation and activation of PDX-1 even at low glucose concentrations, with a final increase in insulin mRNA levels [97]. However, NaW increased basal insulin release, without affecting glucose-induced insulin secretion, in a partially p38-dependent manner [97]. These differences may be explained by the differences in treatment duration, NaW concentrations and cell models used in each study.

#### Summary

NaW promotes β-cell survival through induction of proliferation and inhibition of apoptosis, and also enhances insulin production, which is attributable to activation of PDX-1. NaW effects on β-cell were dependent on MAPK (p38 and ERK) and PI3K activation.

### Adipose tissue

Normal fuel homeostasis involves reciprocal regulation of glucose and lipid catabolism, in a way that glucose availability inhibits fatty acid oxidation and promotes fatty acid synthesis, mainly commanded by insulin signaling [99]. During obesity and insulin resistance, glucose uptake is impaired and enhanced fatty acid oxidation supplies intracellular energy, whereas release of free fatty acids into the circulation is increased for direct utilization by other organs [100]. NaW acts on adipocyte metabolism in an IR-independent manner [101]. NaW induced up-regulation of GLUT4 mRNA expression and increased glucose transport and consumption [102]. NaW treatment did not alter proliferation or viability, but it decreased triglyceride accumulation, which was attributed to a reduced gene expression of enzymes involved in fatty acid synthesis and lipogenesis, such as lipoprotein lipase (LPL), adipocyte protein 2 (aP2), acetyl-CoA carboxylase (ACC1) and fatty acid synthase (FAS), and also to a shift in the mRNA expression profile of transcription factors involved in adipocyte differentiation, with a decreased expression of CCAAT/ enhancer-binding protein (C/EBP)-α and PPARγ2 and increased expression of C/EBP-β [42]. Moreover, NaW induced an increase in mRNA levels of both liver-enriched activator protein (LAP) and liver-enriched inhibitory protein (LIP) isoforms of C/EBP-β, but an overall increment in the LIP/LAP ratio explained the decreased adipogenesis [42]. NaW effect on triglyceride accumulation and C/EBP-β expression was dependent on ERK1/2, but not p38 or PKB/Akt activation [42], positioning the MAPK pathway again as a key signaling cascade for NaW actions in adipocytes. Moreover, NaW stimulated oxygen consumption both through coupled and uncoupled respiration in cultured adipocytes, but without affecting mRNA expression of key oxidative genes, such as PPARα, muscle-carnitine palmitoyltransferase-1 (mCPT-1), medium chain acyl CoA dehydrogenase (MCAD), cytochrome C oxidase subunit I (CO-I) or uncoupling protein (UCP)-2 [42]. *In vivo*, oral administration of NaW to high-fat diet-induced obese rats significantly decreased body weight gain and adiposity without modifying caloric intake, intestinal fat absorption, or growth rate [31]. NaW induced a reduction of white adipose tissue mass through apoptosis and reduction in the size of adipocytes [31]. In contrast to cultured adipocytes, NaW upregulated expression of UCP-1 in brown adipose tissue [30,31,103] and UCP-2 in white adipose tissue [31], favoring thermogenesis as fatty acid oxidation. This increased expression, at least for UCP-1 in brown adipose tissue, was attributed to NaW-induced upregulation of PGC1α [30,103], a transcription co-activator involved in regulation of thermogenesis. Notably, NaW treatment also upregulated gene expression of LPL, aP2, and mCPT-1, but did not affect fatty acid translocase (FAT/CD36) or acyl-CoA oxidase (ACO) in white adipose tissue, indicating that NaW increases fatty acid oxidation but limits an excessive production of ROS [31]. At this regard, it has also been shown that, at the protein level, NaW induced up-regulation of aldehyde dehydrogenase and down-regulation of aldehyde reductase in white adipose tissue [104], and also up-regulation of chaperones (GRP75/hsp70 and CH60), catalase, transketolase and aldehyde dehydrogenase in brown adipose tissue [103] from obese rats, suggesting an overall improvement in the generation of ROS in adipocytes. Indeed, NaW was able to reverse obesity-induced protein oxidation [103]. Moreover, proteins involved in lipid metabolism (monolgyceride lipase, fatty acid binding protein, apolipoprotein A4 and A1, and cholesterol esterase) and cellular structure/cytoskeleton organization (lamin, annexin, vimentin and vinculin) were up-regulated in white adipose tissue from obese rats, and NaW treatment was able to normalize the expression (except for vinculin, which was further enhanced after NaW treatment), explaining restoration of metabolism, and decreased remodeling/differentiation of adipocytes [31,104]. Besides, NaW treatment induced up-regulation of proteins involved in signal transduction, especially Raf-1 kinase, which in turn activates the ERK pathway [104].

Interestingly, NaW anti-obesity effects were dependent on an intact leptin signaling pathway since treatment of obese leptin receptor-deficient (fa/fa) Zucker rats or obese leptin-deficient (ob/ob) mice did not result in reduced body weight gain and food intake, or increased energy expenditure [30]. Nevertheless, transplantation of adipose tissue from lean mice in ob/ob mice, or treatment with recombinant leptin restored the NaW-induced actions, despite NaW did not enhance leptin blood levels [30]. Notably, NaW did not alter expression of UCP-1, PGC1α or CO-I in brown adipose tissue from ob/ob mice, which is in agreement with the fact that leptin regulates (at least) UCP-1 expression by acting centrally through the sympathetic innervation and not directly on brown adipose tissue [30]. These data also explain the absence of effect of NaW on the expression of key oxidative genes in cultured adipocytes [42].

#### Summary

NaW is a potent anti-obesity agent, reducing weight gain and food intake, and increasing oxygen consumption and thermogenesis in obese animal models in a leptin-dependent fashion. ERK1/2, but not PKB/Akt activation, is necessary at least to explain NaW-mediated inhibition of triglyceride accumulation and decreased adipogenesis.

### Brain

The brain energy homeostasis also suffers the consequences of metabolic imbalances, such as insulin resistance, hyperglycemia, and obesity, affecting in turn the feeding behavior and energy expenditure [105]. NaW is able to cross the blood-brain barrier, reaching a concentration of 1.3mg/L (4µM) in the cerebrospinal fluid 30 min after intra-peritoneal injection in rats [44]. Normalization of creatine kinase activity [106], reduction of ROS and oxidized proteins levels, and enhancement of antioxidant power [107] has been observed in the brain from NaW-treated STZ-diabetic rats, suggesting a restoration of brain metabolism. Also, NaW induced GSK3β phosphorylation in brain from insulin-resistant rats and in the human neuroblastoma SH-SY5Y cell line [108]. Since GSK3β is recognized as a key factor in the insulin signaling pathway, it is expected that NaW-induced GSK3β inactivation alleviates brain insulin resistance. At this regard, insulin resistance has been associated with neurodegenerative disorders, including Alzheimer's disease [109]. The microtubule-associated protein Tau is a well-known substrate of GSK3β in neurons, and hyperphosphorylation of Tau has been involved in Alzheimer's disease [110]. NaW-dependent inactivation of GSK3β and subsequent dephosphorylation of tau protein (through the ERK1/2, but not PI3K signaling) opens a new possibility for NaW use in the treatment of this neurological disorder [108,110]. Leptin is another important hormone involved in energy homeostasis, mainly secreted by the adipose tissue in correlation with fat content, and defects in the genes encoding either leptin or its receptor, or resistance to the action of this hormone, lead to obesity [111]. Leptin binds to its receptors in the arcuate nucleus of the hypothalamus, inhibiting neurons that express orexigenic neuropeptide Y (NPY) and agouti-related peptide (AgRP), and activating neurons that express anorexigenic proopiomelanocortin (POMC) and cocaine- and amphetamine-related transcript (CART) [105]. Notably, in lean, but not in leptin-deficient ob/ob mice, NaW decreased NPY/AGRP, and increased CART gene expression, without altering POMC [30]. A decrease in food intake and body weight gain was observed 24 hs after injection of NaW directly in the third cerebro-ventricular cavity, the same effect produced by leptin injection [44]. As leptin, NaW induced phosphorylation of Janus kinase (JAK)-2 and the consequent phosphorylation of Signal transducer and activator of transcription (STAT)-3 on Tyr705, and also phosphorylation of ERK1/2 and, subsequently of STAT3 on Ser727, both in vivo in rat hypothalamus and in vitro in the hypothalamic cell line N29/4 [44]. In N29/4 cells, NaW increased mRNA levels of c-fos and c-myc (STAT3 targets) as soon as 30 min after treatment, and decreased mRNA expression of AgRP but not NPY. Interestingly, JAK2 activation was necessary for leptin- and NaW-induced phosphorylation of STAT3 on Tyr705, but not for NaW-induced phosphorylation of STAT3 on Ser727. Moreover, JAK2 activation was required for leptin- and NaW-induced up-regulation of c-myc gene expression, but no for NaW-induced up-regulation of c-fos. Neither AgRP nor NPY expression was affected by JAK2 inhibition [44]. On the contrary, inhibition of ERK pathway at the level of MEK, decreased phosphorylation of STAT3 on Ser727, but had no effect on phosphorylation of STAT3 on Tyr705, despite leptin or NaW treatment. In addition, up-regulation of c-fos but not c-myc was inhibited this time, together with impaired down-regulation of AgRP after either of both treatments [44].

Excessive caloric intake has been proposed as a negative signal for remodeling of neuronal networks in the adult hypothalamus, while caloric restriction potentiates remodeling of brain areas, suggesting that neuronal plasticity is important for a correct energy balance [112,113]. NaW treatment has shown to alter expression of proteins in arcuate (ARC), lateral (LHA) and paraventricular (PVN) nuclei in the hypothalamus from both lean and obese mice [28]. Expression of proteins involved in cytoskeleton structure (alpha-internexin (INA), neurofilament medium polypeptide (NEFM), glial fibrillary acidic protein (GFAP), βactin, α and β tubulin) and remodeling (collapsin response mediator protein (CRMP)-2) was influenced by NaW [28]. Interestingly, neurofilament proteins are regulated through MAPK and STAT3 [114], and CRMP-2 through PI3K, MAPK and GSK3β [115], which in turn have been shown to be modulated by NaW [25,27,28,44,108]. In the ARC nucleus, NaW induced an increase in the number of c-fos positive cells, which has been previously shown to be mediated by activation of the MAPK pathway [44], and also in number and size of GFAP-positive astroglia [28]. A reduction of β tubulin and syntaxin 1 (HPC-1), and an increase of the neurogenic differentiation (NeuroD)-1 transcription factor and synaptosomal-associated protein (SNAP)-25 expression was also detected in the ARC nucleus from NaW-treated mice [28]. Notably, in vivo magnetic resonance studies showed that NaW treatment had an impact on PVN, but not ARC nucleus' microstructure, probably due to a higher degree of neuronal organization [28].

#### Summary

NaW can act as a leptin-mimetic compound in vivo, correcting feeding behavior. Moreover, it induces neuronal plasticity and remodeling of hypothalamic nuclei, maybe modifying the communication between them.

### Intestine

Chronic hyperglycemia during insulin resistance and diabetes is due to impaired glucose extraction from the blood, to enhanced endogenous glucose production and release from gluconeogenic tissues, and to increased glucose absorption at the intestinal level [116]. STZ-diabetic rats showed increased activity of intestinal sucrase, affecting the final amount of glucose available for absorption [117,118]. Moreover, over-expression at the protein, but not mRNA level, of Na+/glucose co-transporter (SGLT)-1, the main transporter involved in glucose uptake by the small intestine, accounted for enhanced glucose transport [118]. Notably, NaW treatment has shown to normalize both sucrase activity and SGLT-1 expression and activity in diabetic but not in control rats, explaining the glucose lowering effect [118]. Besides, NaW has shown to reduce oxidative stress in the intestinal mucosa from cirrhotic rats [119]. Given that oxidative stress is a well-known mediator of hyperglycemia-induced damage, NaW may be involved in restoration of redox balance in the intestine from diabetic animals.

#### Summary

NaW restores sucrase and SGLT1 activity in small intestine, reducing the amount of glucose absorption in diabetic rats. It is also involved in anti-oxidant mechanisms of intestinal mucosa.

### Reproductive system

It is well-known that diabetes impairs reproductive functions, not only because chronic hyperglycemia leads to glucotoxicity and micro/ macrovascular diseases, but also because insulin signaling itself is needed for a proper activity of reproductive organs [120]. On one hand, ovary function and estrous cycle in females, and on the other hand, testosterone synthesis and total number of Leydig cells in males are altered, and therefore fertility declines in diabetic animal models [121,122]. Diminished serum levels of follicle-stimulating (FSH) and luteinizing (LH) hormones, and consequently, impaired ovarian production of estrogen and progesterone, and testicular production of testosterone, are also detected [37,38]. NaW-treated male and female STZ-diabetic rats showed a recovery of FSH and LH blood levels, together with an increase in libido and fertility [37,38]. In males, NaW recovered the number of Leydig cells and serum testosterone levels, both indirectly by normalizing glucose, insulin and FSH/LH levels, and directly by increasing IR expression and activating MAPK pathway and inactivating GSK3β [37]. In females, protein levels of estrogen (ER) and progesterone (PR) receptors were increased in ovary, and decreased in uterus from diabetic rats, and NaW treatment completely restored ER but partially restored PR expression only in ovary. Contrary to ER, NaW did have a negative effect on PR expression in the non-diabetic ovary [38]. Moreover, decreased mRNA expression of FSH and LH receptors was detected in both ovary and uterus from diabetic rats, but NaW treatment only normalized FSH receptor expression. Ovaries and uterus from diabetic rats expressed higher levels of GLUT3, and NaW was able to normalize them [38], suggesting that normalization of glucose uptake was one mechanism involved in NaW positive effects. Nevertheless, insulin is as a key factor in female reproductive function, and NaW has been shown not to activate the complete insulin signaling pathway in other tissues [25,27,75]. Moreover, NaW was unable to restore depressed serum levels of progesterone in diabetic rats, explaining the incomplete effect of NaW in restoration of reproductive function.

#### Summary

NaW restores FSH and LH serum levels in both male and female diabetic rats, contributing to restoration of estrogen (but not progesterone) and testosterone production, and partial normalization of reproductive function.

### Kidney

The kidney does not only filter the blood and functions as an excretory organ, but it also participates in endogenous glucose production [123]. During diabetes, chronic renal reabsorption of increased amounts of glucose contributes to hyperglycemia [123]. The kidney is an insulin-sensitive organ, and insulin signaling has been reported to be impaired in the diabetic kidney [124]. Despite the kidney is a key organ in the progression of diabetes, little is known about the effects of NaW on its function. One characteristic histopathological feature of the diabetic kidney, the so-called Armanni-Ebstein lesion, is an extensive glycogen deposition in the tubular cells [125]. This hydropic lesion was detected in STZ-diabetic rats, and long-term treatment with NaW was able to completely revert it [46]. The triggering factors of the pathological accumulation of glycogen in the diabetic kidney are not known [126], but it is reasonable to propose that NaW-induced normalization of glycemia is one important mechanism, although a direct effect on renal metabolism cannot be ruled out.

#### Summary

NaW totally reverses the main histopathological lesion of diabetic kidney, i.e. glycogen accumulation in tubules.

## Tungsten vs Vanadium Compounds as Anti-Diabetic Agents. Clinical Trials in Human Patients

It has been shown that vanadium compounds act as insulin-mimetics in liver, skeletal muscle and adipose tissue [127]. However, inorganic vanadium salts have also been associated with undesired side-effects such as gastrointestinal discomfort, severe decrease in body weight gain [128], and liver and kidney toxicity in experimental animal models, independent of the form of vanadium used [129,130]. Some small-scale and short duration trials have proven the beneficial effects of inorganic vanadium salts on both T1D and T2D human patients [131–134]; however, despite much lower doses of vanadium were used than in experimental animals, gastrointestinal discomfort was the most common toxic effect detected in human patients [132,133]. Hence, due to potential toxicity of inorganic vanadium salts at higher doses and long-term therapy in human patients, new organo-vanadium compounds with higher potency have been investigated, showing to be much safer and less toxic, at least regarding gastrointestinal discomfort, and hepatic or renal toxicity [128,135,136]. Unfortunately, the only study with such an organo-vanadium compound (bis (2-ethyl-3-hydroxy-4-pyronato) oxidovanadium(IV)) which has reached preclinical phase II in human patients, was terminated after three months because of renal complications (Akesis Pharmaceuticals, Inc., 2009. http://www.biospace.com/News/akesispharmaceuticals-discontinues-sole-clinical/123583). Contrary to NaW [25], inorganic vanadium salts induce the phosphorylation of the insulin receptor on tyrosine residues [137]. Also, organo-vanadium compounds synergize with insulin to induce further phosphorylation of the insulin receptor [138], which may produce undesired hypoglycemic effects. Moreover, whereas insulin and NaW action on ERK1/2 is transient, vanadate stimulation lasts up to 20 min [139]. Furthermore, vanadium compounds activate PKB/Akt [138,140,141], whereas NaW does not [25,27,42,75,77]. Hence, vanadium salts, either organic or inorganic, are related with a more potent and complete activation of the insulin signaling pathway which may have undesired effects, with special considerations regarding hypoglycemic events due to prolonged activation.

Several patents have applied for the use of NaW in the treatment of different human diseases: non-diabetic obesity (patents WO2002098435 A1; EP1400246 A1), neurodegenerative disorders, particularly Alzheimer's disease and schizophrenia (patents WO2007014970 A1; CN101237875 B), diabetes mellitus (EP0755681 A1), and its use as an anti-platelet agent (WO2014096498 A1). These patents have been published many years ago, some of them have been withdrawn, others lapsed, but only one of them has reached the clinical trial phase. A prospective, randomized, placebo-controlled, double-blind, proof-of-concept trial to address the previously described anti-obesity activity of NaW was carried out in obese patients (http://clinicaltrials.gov, identifier NCT00555074). Thirty moderately obese non-diabetic, mostly normolipidaemic and normotensive patients received 100 mg NaW or placebo twice a day during 6 weeks [142]. Data demonstrated that neither weight/percent of body fat mass, nor energy expenditure/caloric consumption were significant changed by NaW treatment [142]. Differences in biochemical parameters, such as fasting plasma glucose and insulin, lipid profile, leptin and thyroid hormones were not statistically significant between treated and untreated patients either [142]. Besides, no abnormalities in safety parameters (enzymes reflecting liver, muscle and kidney function, and other blood proteins) were detected between the two groups, confirming a low toxicity profile of NaW [143]. The reasons for the differences in the anti-obesity effect of NaW between rodents [28,30,31,42] and humans [142] are not known yet, but they may be attributed to short duration of treatment, to a low dose of NaW, or both. Since NaW acts without any adverse effects such as gastrointestinal discomfort, and given the fact that it was always less toxic than sodium vanadate [143], it is reasonable to propose that organo-tungsten compounds may offer a better and safer way to administer this trace element for use in diabetes therapy. No research has been orientated on this direction so far.

### Summary

- Restoration of normoglycemia and normotrygliceridemia, hallmarks of NaW effects in animal models of metabolic syndrome and diabetes, are mainly achieved by: diminished intestinal glucose absorption; increased glycogenesis and decreased gluconeogenesis in the liver; enhanced glucose uptake by the muscle and adipose tissue; increased number and improved function of β-cells; and decreased lipogenesis and adipogenesis in adipose tissue.
- Anti-obesity effects of NaW are dependent on an intact leptin system.
- All the above-mentioned effects are dependent on ERK1/2 activation by NaW.
- Absent hypoglycemic events of NaW action might be attributed, at least in part, to partial induction of the insulin signaling pathway, and stimulation of β-cell insulin secretion by normal or high, but not low glucose concentrations.

## Conclusion

Nowadays, insulin and different oral hypoglycemic drugs are commercially available; however diabetes mellitus still remains a major health concern for humans. On the basis of all the research articles used for this review, it is reasonable to propose that NaW may offer a potent and complete oral therapy for treatment of T2D patients, and also for those patients who have higher risk of developing diabetes due to overweight, hyperglycemia, and hypertension. Although there is no study addressing co-treatment of NaW with other agents, the use of NaW as a complement for other anti-diabetic drugs may offer new possibilities. Furthermore, NaW may serve as a complement in the treatment of T1D patients with insulin. Its low toxicity profile, together with the great benefits reported in experimental animal models, strongly suggest that NaW deserve more attention to reach a better understanding of the molecular mechanisms involved. Despite all the overwhelming evidence, only one human trial has been performed to date, with neither negative nor positive results. The differences in the effect of NaW between humans and rodents are unknown, and should be further investigated in order to reproduce the anti-diabetic and anti-obesity effects in human patients.
